# Assessing the Incidence of Symptomatic Respiratory Syncytial Virus Illness Within a Prospective Birth Cohort in Managua, Nicaragua

**DOI:** 10.1093/cid/ciz585

**Published:** 2019-07-29

**Authors:** John Kubale, Guillermina Kuan, Lionel Gresh, Sergio Ojeda, Eduardo Azziz-Baumgartner, Nery Sanchez, Roger Lopez, Eva Harris, Angel Balmaseda, Aubree Gordon

**Affiliations:** 1 Department of Epidemiology, School of Public Health, University of Michigan, Ann Arbor; 2 Sócrates Flores Vivas Health Center, Ministry of Health; 3 Sustainable Sciences Institute, Managua, Nicaragua; 4 Centers for Disease Control and Prevention, Atlanta, Georgia; 5 Laboratorio Nacional de Virología, Centro Nacional de Diagnóstico y Referencia, Ministry of Health, Managua, Nicaragua; 6 Division of Infectious Diseases and Vaccinology, School of Public Health, University of California, Berkeley

**Keywords:** respiratory syncytial virus, Nicaragua, cohort study, incidence rate, pneumonia

## Abstract

**Background:**

Respiratory syncytial virus (RSV) causes substantial morbidity and mortality among children worldwide, commonly through acute lower respiratory tract infections (ALRI). To assess the incidence rate of symptomatic RSV illness among young children, we conducted a prospective birth cohort study following children from 0–2 years of age in Managua, Nicaragua.

**Methods:**

Children meeting the testing criteria (fever, history of fever, or severe respiratory symptoms [apnea, stridor, nasal flaring, wheezing, chest indrawing, and/or central cyanosis]) were tested for RSV infections using real-time reverse transcriptase-polymerase chain reaction. An acute lower respiratory infection was defined as a diagnosis of pneumonia, bronchiolitis, bronchitis, or bronchial hyperreactivity. The incidence rate was calculated, and 95% confidence intervals were estimated using a Poisson distribution.

**Results:**

A total of 833 children participated in the cohort: 289 (34.7%) had at least 1 episode of laboratory-confirmed RSV, and 156 (18.7%) of had an episode of RSV-associated ALRI (RSV-ALRI). The incidence rate of symptomatic RSV was 248.1 cases per 1000 person-years (95% confidence interval [CI] 223.2–275.7). While infants aged 6–11 months had the highest incidence of symptomatic RSV (361.3/1000 person-years, 95% CI 304.4–428.8), infants <3 months had the highest incidence of severe RSV (RSV-associated hospitalizations and/or severe ALRI). RSV was also associated with 25.0–37.5% of deaths from medical causes (n = 8).

**Conclusions:**

A substantial burden of RSV exists among children aged <2 years in Nicaraguan communities. RSV was also a leading cause of infant mortality among study participants. The development and implementation of effective RSV prevention and treatment measures represent an opportunity to substantially reduce severe illness and death among children worldwide.

## Teaser

Around one-third of medical deaths among Nicaraguan children under 2 years were respiratory syncytial virus–associated, suggesting it’s an important driver of infant mortality in highly vaccinated populations with little human immunodeficiency virus or malaria.

Respiratory syncytial virus (RSV) is an important cause of acute lower respiratory tract infections (ALRI) like pneumonia and bronchiolitis, particularly among children [1]. In 2015, there were an estimated 33.1 million cases of RSV-associated ALRI (RSV-ALRI) worldwide, of which 3.2 million required hospitalization [2]. This burden is especially pronounced among young children, with an estimated 1.4 million RSV-ALRI hospitalizations and 27 300 in-hospital deaths among infants aged <6 months [2].

Significant disparities exist in the distribution of RSV-associated mortality, with an estimated 99% of in-hospital deaths occurring in low- and middle-income countries (LMICs) [3]. In Nicaragua, severe acute respiratory infections remain the leading communicable cause of death among children aged <5 years [4]. While increasing attention has been given in recent years to improving our understanding of the global burden of RSV, substantial knowledge gaps remain in LMICs. Many studies have used hospital-based populations to study RSV burdens [5–11], but community-based studies are less common [12–16].

Clinically, RSV infection often presents with respiratory symptoms like a cough, rhinorrhea, and difficulty breathing. As many as 97% of children are infected with RSV by age 2 [17]. RSV has also been associated with the development of severe illnesses and is considered the most common viral cause of pneumonia among children aged <5 years [18]. In Nicaragua, the respiratory illness season can last from June through February. While the seasonality of influenza in Nicaragua has been documented [19], the seasonality of RSV and other respiratory viruses is not well-defined.

RSV has long been a target for vaccine development because of its ubiquity and potential for causing severe illness. An overview of RSV vaccines and monoclonal antibodies in development reported 21 candidates in clinical trials [20]. Addressing knowledge gaps about the burden of RSV is crucial to the investment case for these interventions, and their successful future implementation. This study aims to assess the incidence of RSV among young children in Nicaragua, a lower-middle income tropical country in Central America [21]. We used the Nicaraguan Influenza Birth Cohort Study [22], originally designed to examine the incidence of influenza, as it provides a unique opportunity to investigate other respiratory pathogens, such as RSV.

## METHODS

### Ethics Statement

This study was conducted as a collaboration between the Sustainable Sciences Institute, the Nicaraguan Ministry of Health, the University of California, Berkeley (UCB), the University of Michigan, and the US Centers for Disease Control and Prevention (CDC). The study was approved by the Institutional Review Boards (IRBs) of the Nicaraguan Ministry of Health, University of Michigan, and UCB. The CDC’s IRB relied on the UCB IRB for approval. Written informed consent was obtained from a parent/guardian of all participants.

### Study Population

A detailed description of this study has been previously published [22]. The Nicaraguan Influenza Birth Cohort Study was a prospective cohort study conducted year-round from 2011–2016 in the catchment area of the Health Center Sócrates Flores Vivas (HCSFV) in Managua, Nicaragua. Continuous enrollment of newborns was conducted between 8 September 2011 and 5 September 2014 (Supplemental Figure 1). Eligible subjects were identified when brought to the HCSFV for their first well-baby visit, or by home visits. Those who met the enrollment criteria, and for whom informed consent was received, were enrolled into the study. To be included, (1) infants had to be ≤4 weeks of age at enrollment, (2) the family had to live in the HCSFV catchment area, (3) infants’ guardians had to plan to live in the area during the following 2 years, and (4) guardians had to be willing to attend HCSFV for all the infant’s medical visits. Infants who required continued hospitalization directly after birth for ≥4 weeks were not eligible. Enrolled participants remained in the study until their second birthday, they were withdrawn, or they were lost to follow-up.

### Data

Baseline information about demographics, risk factors, and socioeconomic status were collected through surveys conducted by study staff at enrollment and yearly in March/April. Daily symptom diaries were completed by parents and were collected by study staff during weekly home visits. Respiratory samples were collected from infants who met the testing definition by (1) presenting with influenza-like illness, meaning a fever (temperature ≥ 37.8°C) or history of fever and rhinorrhea and/or cough [23]; (2) presenting with fever or history of fever without defined focus; (3) presenting with severe respiratory symptoms (ie, apnea, stridor, nasal flaring, wheezing, chest indrawing, and/or central cyanosis) as judged by a study physician, regardless of the presence of fever/history of fever; or (4) being hospitalized with respiratory symptoms (previously listed) or sepsis [22].

### Sample Collection and Respiratory Syncytial Virus Testing

Oropharyngeal specimens collected with un-flocculated, polyester-tipped plastic swabs (Fisher Scientific, catalog number: 23-400-111) were obtained from infants aged <6 months who met the testing definition, while combined nasal and oropharyngeal swabs were collected from infants aged ≥6 months. Laboratory testing for RSV was conducted by the National Virology Laboratory at the National Center for Diagnosis and Reference of the Nicaraguan Ministry of Health, which has demonstrated proficiency in RSV testing through CDC–Quality Control for Molecular Diagnostics External Quality Assessment [24]. RNA was extracted (QIAamp Viral RNA Mini Kit, Qiagen) and tested by real-time reverse transcriptase-polymerase chain reaction for RSV using CDC protocols [25].

### Clinical Definitions

Clinical care was provided to all study participants at the HCSFV by study personnel, and data were collected for each encounter, regardless of the reason for the visit. Laboratory-confirmed cases of RSV were classified as symptomatic RSV illnesses. Samples positive for RSV occurring ≥14 days from symptom onset for a previous RSV illness were considered new illness episodes. A symptomatic RSV illness was further classified as ALRI (RSV-ALRI) if study physicians diagnosed an acute illness affecting the lower respiratory tract (ie, pneumonia, bronchiolitis, bronchitis, or bronchial hyperreactivity). Pneumonia diagnoses were made by study physicians according to the Integrated Management of Childhood Illness guidelines [26]. Severe ALRI was used instead of severe pneumonia, as done by Shi et al [2]. Cases of ALRI, severe ALRI, and hospitalization occurring within 14 days of symptom onset of a laboratory-confirmed RSV illness episode were considered to be associated with RSV.

### Statistical Analysis

Person-time was calculated as the number of weeks between the participant’s enrollment and exit from the study (at their second birthday or when withdrawn or lost to follow-up). Infants were not considered to be at risk for the 14 days following symptom onset for an RSV illness episode, and were thus excluded from contributing person-time, except for in measures intended to assess severe RSV (RSV-ALRI, RSV-severe ALRI, and RSV-hospitalization). A Poisson distribution was used to calculate 95% confidence intervals (CIs) for incidence rates. Statistical analyses were conducted using SAS version 9.4 (SAS Institute Inc.). Figures were created using R version 3.4.4 (R Foundation for Statistical Computing).

## RESULTS

Between the start of enrollment in September 2011 and the study conclusion in September 2016, 833 infants were enrolled into the cohort and included in this analysis. The mean follow-up time for participants was 1.7 years (19.9 months; Table 1). A total of 9 (1.1%) infants died during the study, with 8 (88.9%) deaths associated with medical illnesses and 1 (11.1%) resulting from an unknown cause. Over 75% of infants completed the study (n = 629), while 23.4% (n = 195) were withdrawn or were lost to follow-up before study completion. The most common reason infants were withdrawn from the study or were lost to follow-up (60.3%, n = 123) was because the child moved away from the study area. We did not observe any significant differences between the demographics of those who completed the study and those who did not (Supplemental Table 1).

**Table 1.** Characteristics of Study Participants

Characteristic		Total, (N = 833)
Age at enrollment	0–2 weeks	581 (69.8)
	3–4 weeks	249 (29.9)
	5–6 weeks	3 (0.4)
Male	…	415 (49.8)
Mean follow-up time, person-years	…	1.7 (0.6^a^)
Smoking in household	…	249 (29.9)
Mean number of persons in household	…	8.7 (4.4^a^)
Mothers with secondary or tertiary education, n = 830	…	677 (81.3)
Fathers with secondary or tertiary education, n = 810	…	644 (77.3)
Water tap location	Outside	291 (35.0)
	Inside	541 (65.0)
Dirt floor	Yes	94 (11.3)

Data are presented as n (%) unless otherwise indicated.

^a^Standard deviation.

There were a total of 17 209 visits to the study clinic; of these, 15 508 (90.1%) were for acute illnesses. The median number of clinic visits per participant was 18 (interquartile range: 10–30), and 814 (97.7%) participants had at least 1 visit.

### Incidence of Symptomatic Respiratory Syncytial Virus Illness

**Figure 1.**
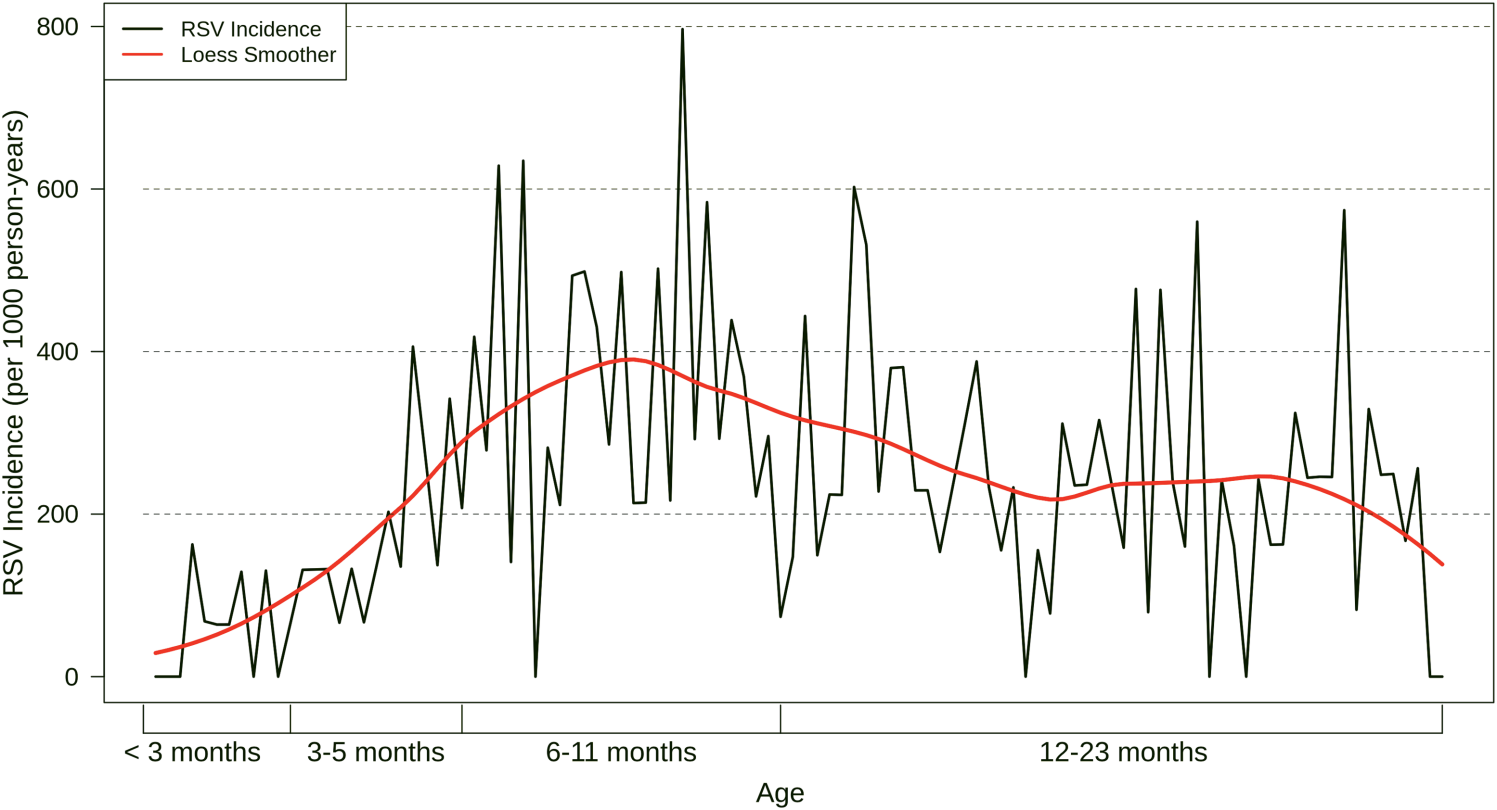
Incidence of symptomatic RSV illness episodes by age. The black line reflects the incidence rate of symptomatic RSV illnesses by week of age, while the red line shows a Loess smoothing function applied to the data to illustrate the overall trend. Abbreviation: Loess, locally estimated scatterplot smoothing; RSV, respiratory syncytial virus.

**Figure 2.**
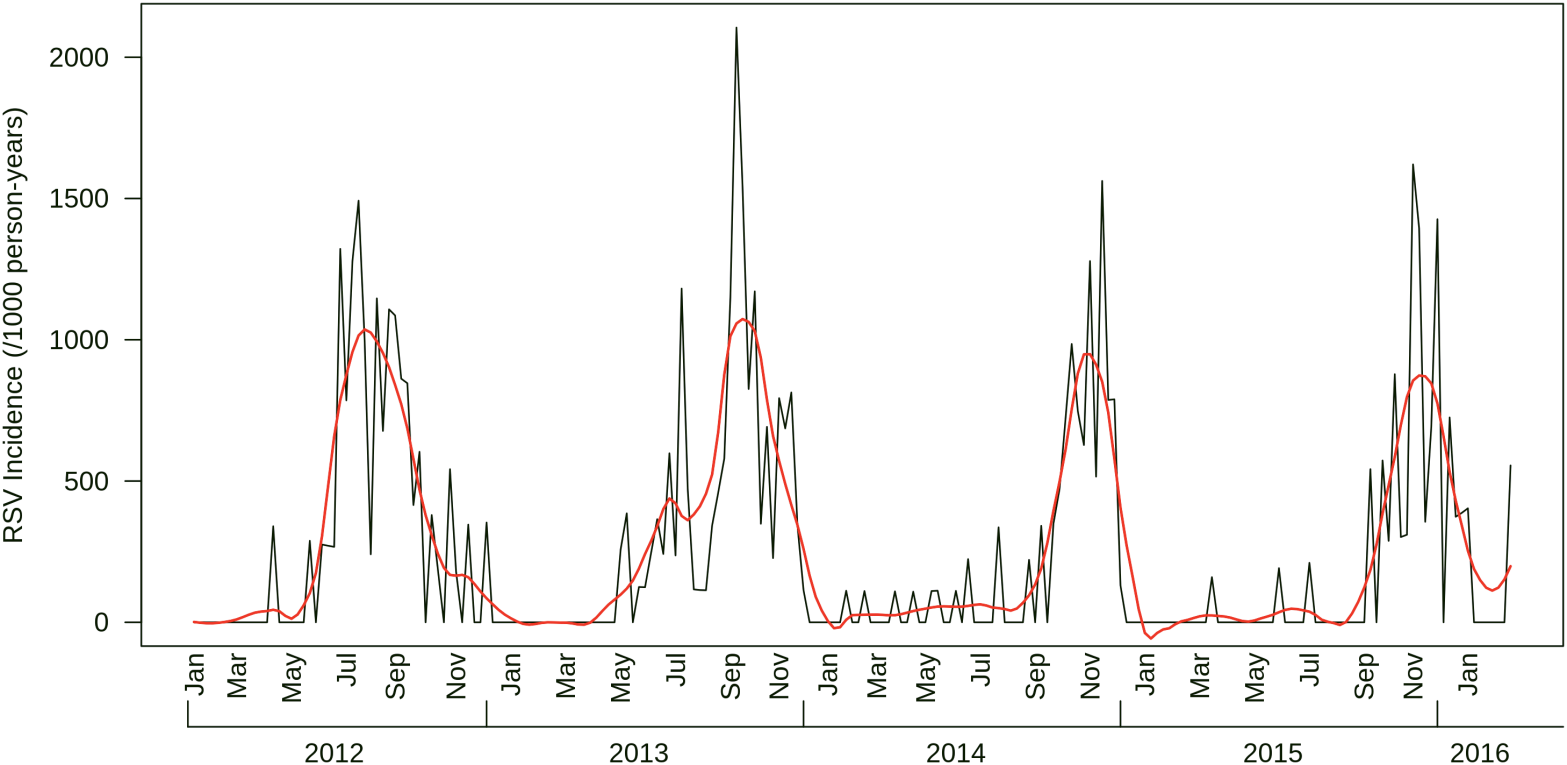
Incidences of symptomatic RSV illness by study week. The black line reflects the incidence rate of symptomatic RSV illnesses by week of study, while the red line shows a Loess smoothing function applied to the data to illustrate the seasonal trend of RSV transmission. Data were truncated at the beginning and end of the study when the total number of participants in the study was below 100. Abbreviation: Loess, locally estimated scatterplot smoothing; RSV, respiratory syncytial virus.

Participants contributed a total of 1417.3 person-years and experienced 344 laboratory-confirmed episodes of symptomatic RSV illness, 11 (3.2%) of which were coinfected with influenza A. We did not observe differential illness severities among those coinfected. Of the 833 infants, 289 (34.7%) had at least 1 documented episode of symptomatic RSV illness. Of these, 50 (17.3%) infants had recurrent (≥2) episodes of symptomatic RSV illness, and 5 (1.7%) experienced 3 episodes of symptomatic RSV illness. The crude incidence of symptomatic RSV illness was 248.1 cases per 1000 person-years (95% CI 223.2–275.7; Table 2). The incidence rate of symptomatic RSV illness increased steadily with age, peaking among infants aged 6–11 months at 361.3 cases per 1000 person-years (95% CI 304.4–428.8), before falling to 249.2 per 1000 person-years (95% CI 214.0–290.1) among those aged 12–23 months (Table 2; Figure 1). There were 176 (51.2%) symptomatic RSV illnesses that did not present with nurse-/physician-measured fever (≥38°C); including measured fever in the symptomatic RSV illness case definition decreased rates by 37–66% (Supplemental Table 2). RSV epidemics started as early as May and as late as September, lasting an average of 6.9 months (range: 4–7 months; Figure 2).

**Table 2.** Incidence of Symptomatic Respiratory Syncytial Virus Illness Episodes

Characteristic		RSV Cases	Person-Years	Incidence Rate (95% CI^a^) Per 1000 Person-Years
All participants		344	1386.8	248.1 (223.2–275.7)
Age	<3 months	10	149.6	66.8 (36.0–124.2)
	3–5 months	37	208.2	177.7 (128.8–245.3)
	6–11 months	131	362.7	361.2 (304.3–428.6)
	12–23 months	166	666.3	249.2 (214.0–290.1)
Sex	Male	176	692.9	254.0 (219.1–294.5)
	Female	168	693.9	242.1 (208.2–281.7)

Abbreviations: CI, confidence interval; RSV, respiratory syncytial virus.

^a^CIs calculated using a Poisson distribution.

### Incidence of Respiratory Syncytial Virus–Associated and Severe Acute Lower Respiratory Tract Infections

**Figure 3.**
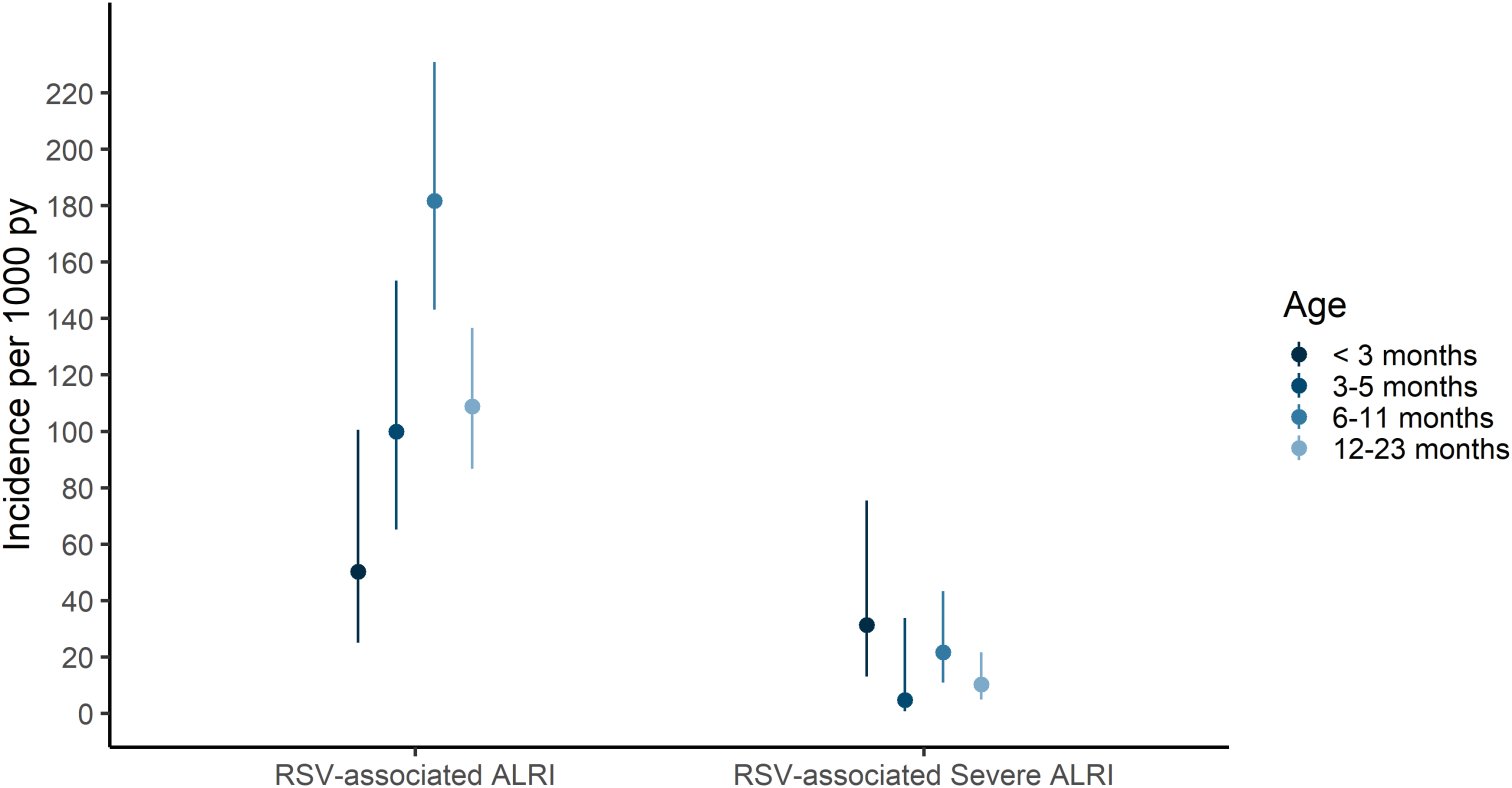
Incidences of RSV-associated ALRIs and RSV-associated severe ALRIs by age. Plot of incidence rates of RSV-associated ALRIs and RSV-associated severe ALRIs by age category. Lines around point estimates represent 95% confidence intervals, estimated using a Poisson distribution. Abbreviations: ALRI, acute lower respiratory tract infections; RSV, respiratory syncytial virus.

Of the 344 laboratory-confirmed cases of symptomatic RSV illness identified in the study, 170 (49.4%) were classified as ALRI (Supplemental Table 3), resulting in an overall incidence rate for RSV-ALRI of 119.9 cases per 1000 person-years (95% CI 103.2–139.4). The incidence rates of RSV-ALRI followed a similar trend across age groups as that of symptomatic RSV illness, with incidences increasing with age until peaking among participants aged 6–11, with 181.8 cases per 1000 person-years (95% CI 143.1–231.0). While children aged <3 months had the lowest overall RSV illness rates, they had the highest rate of RSV-severe ALRI (Figure 3; Supplemental Table 3), though the differences between age groups were not statistically significant.

Among the 170 cases of RSV-ALRI, 21 (12.4%) had severe illnesses (Supplemental Table 3), with an incidence of RSV-severe ALRI of 14.8 cases per 1000 person-years (95% CI 9.7–22.7). Participants aged <3 months had the highest incidence of RSV-severe ALRI, with 31.4 cases per 1000 person-years (95% CI 13.1–75.5). Except for a sharp decline among those 3–5 months of age, the incidences of RSV-severe ALRI decreased as age increased (Figure 3). While episodes of symptomatic RSV illness were less frequent among the youngest participants—aged <3 months—(Table 2; Supplemental Table 3), those that did occur were more likely to be severe. Of children aged <3 months with symptomatic RSV illness, 80% had RSV-ALRI (vs 56.8% among those 3–5 months, 51.2% among those 6–11 months, and 44.6% among those 12–23 months; Chi-square *P* = 0.1); additionally, 50% of children aged <3 months with symptomatic RSV illness had RSV-severe ALRI (vs 2.7% among those 3–5 months, 6.1% among those 6–11 months, and 4.2% among those 12–23 months; Chi-square *P* < .0001).

### Incidence of Respiratory Syncytial Virus–Associated Hospitalizations

The incidence of RSV-associated hospitalizations was 22.6 cases per 1000 person-years (95% CI 16.0–31.9). Aside from a precipitous drop among those aged 3–5 months, incidences of RSV-associated hospitalizations steadily decreased as age increased, with infants aged <3 months having the highest incidence (37.7 cases per 1000 person-years, 95% CI 16.9–83.9; Supplemental Table 4).

### Respiratory Syncytial Virus–Associated Deaths

Of the 8 infants who died from medical causes during the study, 3 (37.5%) died of severe pneumonia (all in-hospital) and were reverse transcriptase-polymerase chain reaction–positive for RSV in the weeks preceding their death. Of these deaths, 2 (25.0%) occurred within 2 weeks (1 and 14 days) of symptom onset; the infant who died 1 day after testing positive for RSV was 4 months old, while the infant who died 14 days after symptom onset was aged 10 months. An additional infant (aged 11 months) died of severe pneumonia 46 days after symptom onset. The RSV-associated mortality rate among infants ranged from 2.8 deaths per 1000 person-years (95% CI .7–11.1) using a 14-day risk period to 4.2 per 1000 person-years (95% CI 1.3–12.9) when considering the deaths that occurred up to 46 days after laboratory confirmation of RSV.

## DISCUSSION

Using data from a community-based, prospective birth cohort study, we found a high incidence of symptomatic RSV illness in Nicaragua in children aged <2 years. Infants aged <3 months had the highest rates of severe RSV infection outcomes, including severe ALRI and hospitalization. In our birth cohort, laboratory-confirmed RSV illness was associated with one-third of deaths. In this population, many common contributors to infant mortality in LMICs are missing, as >98% children received World Health Organization–recommended immunizations [27], the prevalence of human immunodeficiency virus is low [28], and malaria is absent, suggesting RSV is a significant contributor to infant mortality. This finding has important implications for a number of countries that have full coverage under the Expanded Program on Immunization but still struggle to lower infant mortality.

Our findings are consistent with published estimates from other parts of the world [12, 13, 29]. In a review of the 2015 global burden of RSV-ALRI, Shi et al [2] reported incidence rates ranging from 26.6–343.8 per 1000 person-years among children aged 0–5 months, 18.0–338.1 among those aged 6–11 months, and 21.8–304.3 among those aged 12–23 months. A study conducted in the Peruvian highlands was responsible for the highest estimates in all age groups, reporting rates approximately double those of the next highest estimates (Supplemental Table 5) [2, 30]. Our RSV-associated ALRI estimates were similar to the majority of studies referenced by Shi et al [2] (ie, 67, 160, and 93 per 1000 person-years among children aged 0–5, 6–11, and 12–23 months, respectively). We did observe rates of symptomatic RSV and ALRI that peaked later (among infants 6–11 months) than other studies. It is possible that our age-specific estimates of symptomatic RSV are biased from the inclusion of reported/measured fever, as the proportion of RSV illnesses presenting with fever increases with age. However, a study in Guatemala [31] showed a similar pattern, suggesting that regional variations might impact the age distribution of RSV incidences.

There are limited published data about the incidences of RSV in community settings in LMICs, especially in Central America [2]. Shi et al [2] compiled data from 329 studies, of which only 14 (4%) were community-based with active case-ascertainment. Of these 14, only 1 was from Central America (Guatemala) [31]. The Guatemalan study reported an incidence of RSV pneumonia among children aged ≤18 months of 143.6 per 1000 person years (95% CI 116.2–177.3) [31]. While the Guatemala estimate was higher than the estimate in our study (70.6 cases per 1000 person-years, 95% CI 58.0–85.8; Supplemental Table 5), this is likely because, in the Guatemalan study, RSV-ALRI cases were identified only from children with physician-diagnosed pneumonia, not the overall study population.

Identifying and quantifying RSV-associated mortality is challenging, and the most appropriate time period to use in classifying deaths associated with RSV remains a subject of debate [32]. RSV-associated mortality might peak weeks after the original RSV infection and, perhaps, be associated with secondary bacterial infectiosn [33–35]. A recent examination of RSV mortality in Minnesota included deaths that occurred within an 8-week period of laboratory confirmation [36]. Moreover, the quantification of RSV-associated mortality in community-based studies is limited by the fact that only a relatively small number of deaths are expected. However, in our study, out of 8 deaths from medical causes, 2 deaths seemed clearly associated with RSV, because they occurred within 2 weeks of the onset of laboratory-confirmed RSV illness; we could argue that a third death (approximately 6 weeks following RSV laboratory confirmation) was also associated with RSV illness. Thus, 25% or 37.5% of deaths from medical causes were associated with RSV.

This study has a number of strengths. This community-based study provides insight about the largely undocumented burden of RSV in communities in LMICs where a substantial proportion of the population might not seek hospital care for severe illnesses. The study enrolled children from birth and actively monitored them each week throughout the year for respiratory illnesses. As a prospective, longitudinal cohort study, we were able to calculate incidences and examine 4 seasonal RSV epidemics. Finally, by including neonates, we documented RSV rates in a younger age group than much of the existing literature.

Multiple hospital-based studies have demonstrated that the inclusion of fever (measured [≥38°C] or reported) in case definitions results in an underestimate of RSV cases, particularly among children aged <1 year [37–39]. Studies using data from cohorts initially designed to study influenza (like this study) are susceptible to such underestimates, as case definitions like influenza-like illness and severe acute respiratory illness reflect influenza’s more frequent presentation with fever. While we were unable to make direct comparisons across a variety of case definitions—as Saha et al [38], Nyawanda et al [37], and Rha et al [39] did—we did conduct sensitivity analyses examining the effect of including nurse-/physician-measured fevers (≥38°C) on RSV rates. Had a measured fever been included as a required criteria for sampling and/or testing, our estimated incidence rates would have been 30–70% lower, depending on participants’ ages. Such findings suggest the value of developing RSV-specific case definitions, like those pursued through the World Health Organization’s Global RSV Surveillance Pilot [40]. While our testing definition likely missed some cases of symptomatic RSV infection—particularly among those aged <1 year—the majority of any missed cases were most likely among those with less severe illnesses. The inclusion of any severe respiratory symptoms (regardless of fever/history of fever) in this study’s testing criteria suggests that our assessments of more severe manifestations of RSV are good approximations of the true severe RSV burden in our study community. Future studies in this population are underway to examine the specific risks and prognostic factors contributing to this burden.

This study demonstrates that a substantial burden of RSV exists among children aged <2 years in Nicaragua. This, coupled with the high proportion of infant deaths associated with RSV illness, underscores the importance of RSV in such communities. Such findings demonstrate the merit of exploring the cost-benefit of current interventions and of providing continued support for those interventions being developed for pregnant women and young children to prevent RSV illness among these high-risk groups. The development and implementation of effective RSV prevention represents a prime opportunity to substantially reduce the morbidity and mortality of young children in Nicaragua and other LMICs.

## Supplementary Data

Supplementary materials are available at *Clinical Infectious Diseases* online. Consisting of data provided by the authors to benefit the reader, the posted materials are not copyedited and are the sole responsibility of the authors, so questions or comments should be addressed to the corresponding author.

ciz585_Suppl_Supplementary_Material

## Notes

***Acknowledgments.*** The authors thank the study staff at the Health Health Center Sócrates Flores Vivas and the Centro Nacional de Diagnóstico y Referencia for conducting the study, Angela JP Campbell for her helpful comments, and the infants and their families for participating in the study.

***Financial support.*** This work was supported by the US Centers for Disease Control and Prevention (cooperative agreement 1U01GH000028-04) and the National Institutes of Health, Fogarty International Center (grant number K02 TW009483 to A.G.).

***Potential conflicts of interest.*** The authors report no potential conflicts of interest. All authors have submitted the ICMJE Form for Disclosure of Potential Conflicts of Interest. Conflicts that the editors consider relevant to the content of the manuscript have been disclosed.
