# Supplementary material for: Assessing the Incidence of Symptomatic Respiratory Syncytial Virus Illness Within a Prospective Birth Cohort in Managua, Nicaragua
Source: Clin Infect Dis. 2019 Jul 29;70(10):2029–35. doi: 10.1093/cid/ciz585 (PMC7201406; doi:10.1093/cid/ciz585)
Supplement: ciz585_Suppl_Supplementary_Material [file ciz585_suppl_supplementary_material.docx]

**SUPPLEMENTAL TABLES AND FIGURES**

**Supplemental Figure 1. Plot of categorical age distribution over the course of the study.**  Count of enrolled participants by age category across study duration.


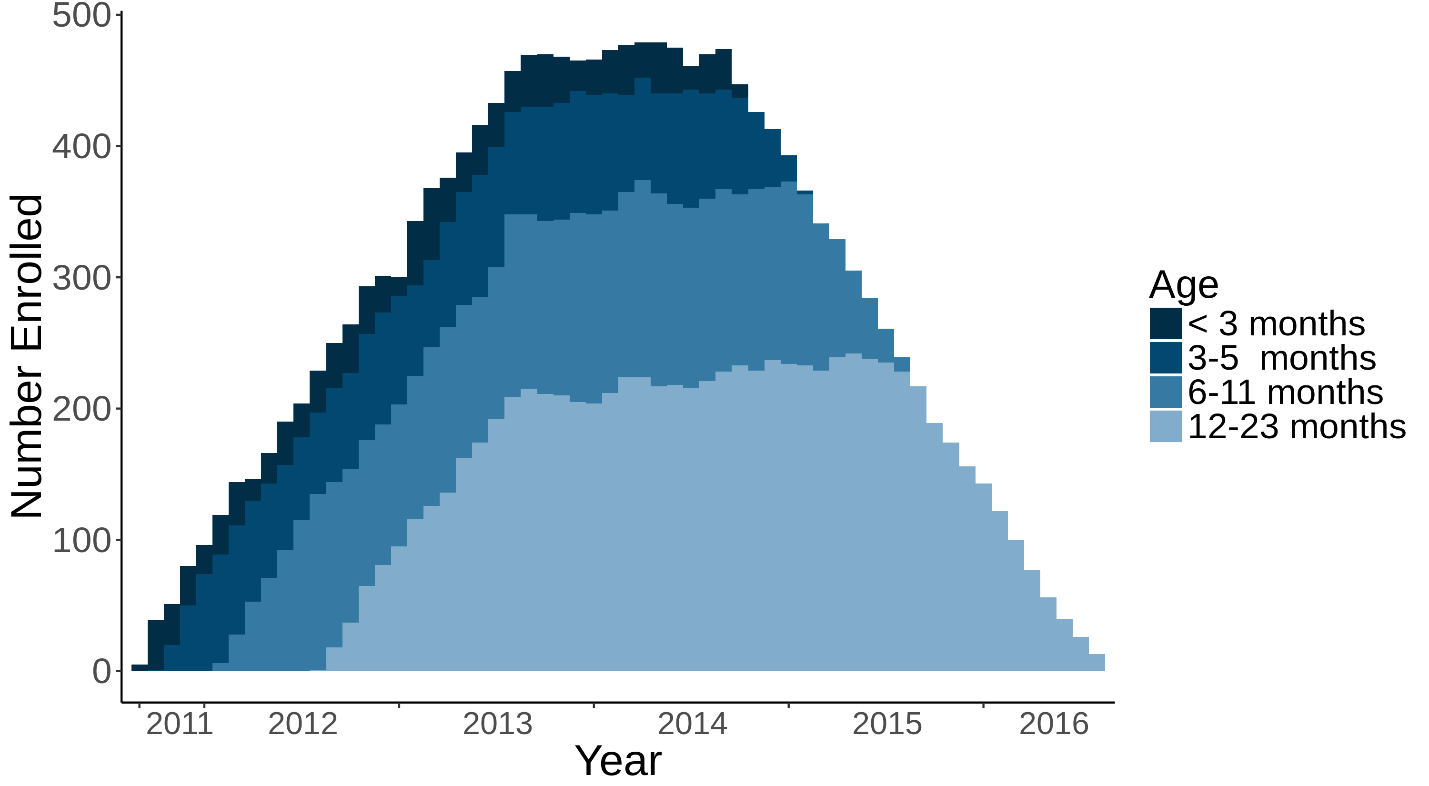


| **Supplemental Table 1. Characteristics of study participants by completion status** | | | | | |
| --- | --- | --- | --- | --- | --- |
| Characteristics | | Total | Completed study | Withdrawn/Lost to follow-up | p-value |
|  | No. | 833 | 629 | 204 |  |
| Age at enrollment | 0-2 weeks | 581 (69.8) | 442 (70.3) | 139 (68.1) | 0.209 |
|  | 3-4 weeks | 249 (29.9) | 186 (29.6) | 63 (30.9) |  |
|  | 5-6 weeks | 3 (0.4) | 1 (0.2) | 2 (0.98) |  |
| Sex | Male | 415 (49.8) | 311 (49.4) | 104 (51.0) | 0.747 |
|  | Female | 418 (50.2) | 318 (50.6) | 100 (49.0) |  |
| Mean person-years contributed |  | 1.7 (0.6^a^) | 1.9 (0.05^a^) | 1.2 (0.5^a^) | <0.001 |
| Smoking in household | Yes | 249 (70.1) | 188 (29.9) | 61 (29.9) | 0.997 |
|  | No | 584 (29.9) | 441 (70.1) | 143 (70.1) |  |
| Mean number in household | | 8.7 (4.4^a^) | 8.7 (4.4^a^) | 8.85 (4.2^a^) | 0.337 |
| Mothers with secondary or  tertiary education | | 677 (81.3) | 519 (82.6) | 160 (79.2) | 0.192 |
| Fathers with secondary or  tertiary education | | 644 (77.3) | 487 (81.3) | 159 (82.4) | 0.878 |
| Data are presented as No. (%) unless otherwise indicated.  ^a^Standard deviation | | | | | |

| **Supplemental Table 2. Sensitivity analysis of RSV incidence when measured fever (≥38°C) was used in testing criteria** | | | | | |
| --- | --- | --- | --- | --- | --- |
| Characteristic | | RSV cases | Person-years | Incidence rate (95% CI^a^) per 1000 person-years | % difference^b^ |
| All participants | | 168 | 1386.8 | 121.2 (104.2, 140.9) | 51.2% lower |
| Age | <3 months | 5 | 149.6 | 33.4 (13.9, 80.3) | 50.0% lower |
|  | 3-5 | 12 | 208.2 | 57.6 (232.7, 101.5) | 70.3% lower |
|  | 6-11 months | 46 | 362.7 | 126.8(95.0, 169.3) | 64.1% lower |
|  | 12-23 months | 105 | 666.3 | 157.6 (130.2, 190.8) | 36.8% lower |
| Sex | Male | 87 | 692.9 | 125.6 (101.8, 154.9) | 50.1% lower |
|  | Female | 81 | 693.9 | 116.7 (93.9, 145.1) | 51.8% lower |
| ^a^Confidence intervals calculated using a Poisson distribution.  ^b^When compared to incidence rates calculated from testing criteria using fever or history of fever (Table 2). | | | | | |

| **Supplemental Table 3. Incidence of RSV-associated ALRI and RSV-associated severe ALRI by age and sex** | | | | | | |
| --- | --- | --- | --- | --- | --- | --- |
| Characteristics | | Person-years | RSV-ALRI | Incidence rate  (95% CI) | RSV-Severe ALRI | Incidence rate (95% CI) |
| All participants | | 1417.4 | 170 | 119.9 (103.2, 139.4) | 21 | 14.8 (9.7, 22.7) |
| Age | <3 months | 159.2 | 8 | 50.3 (25.1, 100.5) | 5 | 31.4 (13.1, 75.5) |
|  | 3-5 months | 210.0 | 21 | 100.0 (65.2, 153.4) | 1 | 4.8 (0.7, 33.8) |
|  | 6-11 months | 368.6 | 67 | 181.8 (143.1, 231.0) | 8 | 21.7 (10.9, 43.4) |
|  | 12-23 months | 679.7 | 74 | 108.9 (86.7, 136.7) | 7 | 10.3 (4.9, 21.6) |
| Sex | Male | 708.3 | 96 | 135.6 (111.0, 165.6) | 12 | 16.9 (9.6, 29.8) |
|  | Female | 709.1 | 74 | 104.4 (83.1, 131.1) | 9 | 12.7 (6.6, 24.4) |
|  | | | | | | |

| **Supplemental Table 4. Incidence of RSV-associated hospitalization and RSV-associated pneumonia by age and sex** | | | | | | |
| --- | --- | --- | --- | --- | --- | --- |
| Characteristics | | Person-years | RSV-associated hospitalizations | Incidence rate (95% CI) | RSV-associated pneumonia | Incidence rate (95% CI) |
| All participants | | 1417.4 | 32 | 22.6 (16.0, 31.9) | 100 | 70.6 (58.0, 85.8) |
| Age | <3 months | 159.2 | 6 | 37.7 (16.9, 83.9) | 6 | 37.7 (16.9, 83.9) |
|  | 3-5 months | 210.0 | 1 | 4.8 (0.7, 33.8) | 9 | 42.9 (22.3, 82.4) |
|  | 6-11 months | 368.6 | 12 | 32.6 (18.5, 57.3) | 42 | 114.0 (84.2, 154.2) |
|  | 12-23 months | 679.7 | 13 | 19.1 (11.1, 32.9) | 43 | 63.3 (46.9, 85.3) |
| Sex | Male | 708.3 | 18 | 25.4 (16.0, 40.3) | 55 | 77.7 (59.6, 101.2) |
|  | Female | 709.1 | 14 | 19.7 (11.7, 33.3) | 45 | 63.5 (47.4, 85.0) |
|  |  |  |  |  |  |  |

| **Supplemental Table 5. Comparison of incidence of RSV-ALRI across studies** | | | | | |
| --- | --- | --- | --- | --- | --- |
| Location/reference | Incidence rate of RSV-ALRI  per 1000 person-years (95% CI) | | | Minimum criteria for sample collection/testing | Study design and population |
|  | 0-5 months | 6-11 months | 12-23 months |  |  |
| **Nicaragua**  **[this study]** | **78.6** | **187.2** | **108.9** | **Fever (≥37.8°C), reported fever, or severe respiratory symptoms** | **Prospective birth cohort study of children 0-2 years, enrolled from home or during well baby visits** |
| Peru [29] | 343.8 | 338.1 | 304.3 | Reported fever or cough in last 7 days | Prospective cohort study including children aged <3 years, recruited from home |
| Kenya [12,13] | 147.0 | 63.0 | 71.0 | Difficulty breathing, rhinorrhea and/or nasal congestion, or cough in last 7 days | Prospective birth cohort over 4 calendar years, recruited from maternity ward or maternal child clinic |
| Dhaka,  Bangladesh [40] | 150.0 | 110.0 | 120.0 | Fever (≥38°C), difficulty breathing, cough, or rhinorrhea | Prospective birth cohort of children 0-2 years, recruited from area surrounding clinic |
| Ballabgarh,  India [28] | 60.2 | 18.0 | 52.0 | Cough or difficulty breathing | Prospective birth cohort of children 0-3 years, recruited from 2 towns following identification from medical records |
| San Marcos, Guatemala [30] | 107.7 | 172.4 | 124.5 | Children diagnosed with pneumonia | Randomized control trial of children ≤18 months comparing homes with traditional or intervention cook stove |
| Table adapted from review paper by Shi et al. [2] | | | |  |  |
